# Whole-Genome Sequencing Identifies Functional Genes for Environmental Adaptability in Chinese Geese

**DOI:** 10.3390/ani15101395

**Published:** 2025-05-12

**Authors:** Xufang Ren, Jincheng Yu, Xiurong Zhao, Xinye Zhang, Gang Wang, Xiaoyu Jiang, Xianyao Li, Changqing Qu, Lujiang Qu

**Affiliations:** 1 Engineering Technology Research Center of Anti-Aging Chinese Herbal Medicine of Anhui Province, Biology and Food Engineering School, Fuyang Normal University, Fuyang 236037, China; rxf1828@163.com; 2 National Engineering Laboratory for Animal Breeding, College of Animal Science and Technology, China Agricultural University, Beijing 100193, China; zxiurong_feign@163.com (X.Z.); xinye_leaf@163.com (X.Z.); wanggang@cau.edu.cn (G.W.); jxy1581908148@163.com (X.J.); 3 Liaoning Academy of Agricultural Sciences, Shenyang 110161, China; yujincheng_pi@126.com; 4 College of Animal Science and Technology, Shandong Agricultural University, Taian 271018, China; xyli@sdau.edu.cn

**Keywords:** environmental adaptability, genetic–environment association, goose, whole-genome sequence

## Abstract

China is home to a rich diversity of goose genetic resources, with more than 30 distinct breeds. These breeds are widely distributed in various environments with high annual mean temperature differences ranging from about 3.5 °C to 23 °C and annual precipitation differences ranging from about 230 mm to 2200 mm, positioning them as excellent models for studying environmental adaptability. With three genetic–environment association methods, the LFMM, Samβada, and RDA analysis, a total of 447 genes were selected by at least two methods. These genes are significantly enriched in three pathways, including the polycomb repressive complex pathway, the alanine, aspartate, and glutamate metabolism pathway, and the GnRH signalling pathway. The scanning of all candidate single-nucleotide polymorphism variations found that only two variants located in a non-coding region (chr3:38968547, chr13:23863699) showed stronger positive selection in breeds living in a high-temperature environment. This research not only deepens our understanding of the genetic basis of goose adaptation to diverse environments but also provides a valuable resource for future selective breeding programs in the goose industry facing rapid global climate change.

## Simple Summary

Rapid and unprecedented global climate change is posing a significant and multifaceted threat to biodiversity. Investigating the genetic footprints of temperature and precipitation selection can provide insights into local adaptation and is helpful for providing strategies to respond to climate change. As the largest goose-producing country in the world, China accounts for more than 70% of global goose breeds. However, few studies have comprehensively demonstrated the genetic signatures associated with environmental adaptation in Chinese geese. To elucidate the environmental adaptability of the Chinese goose, we used three genetic–environment association analysis methods and identified a range of functional genes with temperature and precipitation based on the whole-genome sequencing data of 239 geese collected from 21 breeds. A total of 447 genes were identified as being associated with environmental adaptation, such as the *FAF1* and *RSF1* genes, and genes belonging to the solute carrier family and the nicotinamide adenine dinucleotide hydride (NADH) family. Two variants (chr3: 38968547, chr13: 23863699) located in a non-coding region were identified and exhibited stronger signatures for positive selection in the breeds living in higher temperatures.

## 1. Introduction

One important link of animals during domestication is adaptation [1]. With the combined action of artificial selection and environmental drive, organisms will consciously make new mutations in the direction of their survival [2]. Pressures from different ecological environments have been considered an important promoter in forming different phenotypes. Understanding the genetic basics of adaptation to different environments is a key focus of evolutionary biology, and for poultry breeds and other livestock species, it can provide valuable information for providing breeding strategies under climate change.

Chinese local geese are believed to have originated from two wild ancestors, the swan goose (*Anser cygnoides*) and graylag goose (*Anser anser*). Except for the Yili goose, which descends from swan geese, other domestic geese are primarily derived from graylag geese [3]. An early stage of goose domestication in China can be traced back to approximately 7000 years ago [4]. By 2010, a total of 30 breeds had been certified [5]. These breeds are widely distributed in various environments with annual mean temperature differences ranging from about 3.5 °C to 23 °C and annual precipitation differences ranging from about 230 mm to 2200 mm, which makes them excellent models to study environmental adaptability. In order to adapt to different environments, geese from different regions showed phenotypic differences. For example, in order to resist the cold and store energy, the ZI goose, which lives in the cold region of northeast China, has a larger body weight, developed breast muscles, and thicker subcutaneous fat than those geese living in warm environments.

The exploration of identifying functional markers associated with different environments, including chicken, pigs, goats, and more, has continued [6,7,8,9,10,11]. It is certain that, regardless of the species, adaptation to the environment is controlled by multiple genes. Moreover, candidate genes always have specific variants, and the expression levels of these genes or allele frequencies vary significantly in different environments. Compared to other important livestock and poultry breeds, there is a paucity of genomic studies elucidating the environmental adaptability of geese. A previous study based on selective signature analysis identified a number of genes related to cold adaptation in Huoyan geese, and these genes were mainly involved in physiological functions such as metabolism, angiogenesis contraction and circulatory system, apoptosis, immunity, stress, and the neural system [12]. However, traditional selection signal analysis focuses on inferring the role of natural selection by detecting genetic variation patterns in the genome, usually without directly considering environmental factors.

In this study, the whole genomes of 239 geese from 21 breeds in various temperature and precipitation environments were obtained. The genetic diversity, population structure, and evolutionary history of these Chinese local geese were elucidated. Then, we performed genetic–environment association analysis according to their genomic data, including the latent factor mixed models (LFMM), Samβada, and redundancy analysis (RDA). Finally, candidate genes and pathways that were potentially associated with various adaptations to environments were identified.

## 2. Materials and Methods

### 2.1. Ethics Statement

This study was approved by the Animal Welfare Committee of China Agricultural University (No: AW01405202-1-03), and all geese used in the study were taken care of according to relevant regulations.

### 2.2. Sampling

**Figure 1:**
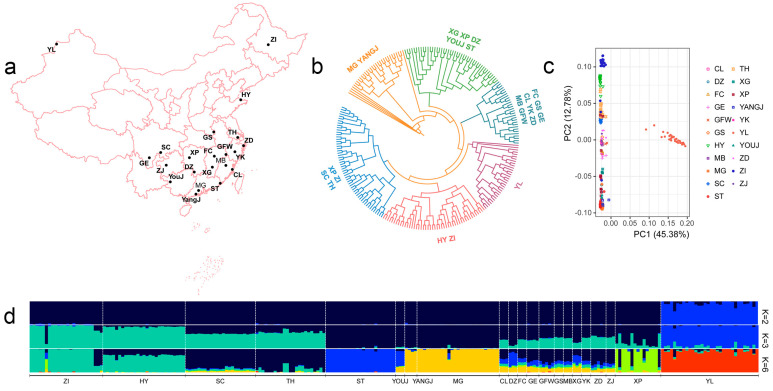
(**a**) Distribution of 21 Chinese local breeds. The abbreviation of goose breeds can be found in Supplementary file S1: Table S1. (**b**) Phylogenetic tree of all samples in this study. (**c**) PCA plot of all samples in this study. (**d**) Admixture results for K = 2, 3, and 6. Colors represent different ancestry.

A total of 239 Chinese domestic geese from 21 breeds (Figure 1a), including 89 newly sequenced and 150 geese downloaded from a public database [13] were used in this study (Supplementary file S1: Table S1). Whole-blood samples were obtained from 89 geese, including 18 geese breeds. Sixteen breeds were from Southern China, including Changle geese (CL, *n* = 3), Daozhou grey geese (DZ, *n* = 3), 3 Fengcheng grey geese (FC, *n* = 3), Gang geese (GE, *n* = 4), Guangfeng White geese (GFW, *n* = 5), Gushi geese (GS, *n* = 3), Minbei white geese (MB, *n* = 3), Magang geese (MG, *n* = 3), Shitou geese (ST, *n* = 3), Taihu geese (TH, *n* = 3), Xingguo Grey geese (XG, *n* = 3), Yangjiang geese (YANGJ, *n* = 3), Yongkang Grey geese (YK, *n* = 3), Youjiang geese (YOUJ, *n* = 3), Zhedong White geese (ZD, *n* = 5), and Zhijin White (ZJ, *n* = 3) geese. Two breeds were from Northern China, including Yili geese (YL, *n* = 32) and Huoyan geese (HY, *n* = 3). Another 150 geese, including 24 HY geese, 24 MG geese, 20 ST geese, 20 TH geese, 15 Xupu (XP) geese, 23 Sichuan (SC) White geese, and 24 Zi geese, were obtained from one published study (BioProject ID: PRJNA695024, PRJNA695024) [13].

### 2.3. Sequencing and Variant Calling

Genomic DNA was extracted using the standard phenol/chloroform extraction method [14]. The quality and integrity of the extracted DNA were verified using a NanoDrop spectrophotometer (Thermo Fisher Scientific, Wilmington, DE, USA). Paired-end (150 bp) libraries were sequenced and built using the Illumina Novaseq 6000 platform, according to the manufacturer’s instructions. Quality control of raw sequencing data was performed using fastp (v0.23.4) [15] with the default parameter. Clean data alignment, preprocessing of alignments, and variation calling were performed by GTX.CAT (v 2.1.1) (http://www.gtxlab.com/en/product/cat, accessed on 1 March 2024). Chromosome-level goose reference genome was downloaded from a published study (http://gigadb.org/dataset/100789, accessed on 1 February 2024) [16]. Extraction and hard-filtering (QD < 2.0 || QUAL < 30.0 || SOR > 3.0 || FS > 60.0 || MQ < 40.0 || MQRankSum < −12.5 || ReadPosRankSum < −8.0) of raw single-nucleotide polymorphisms (SNPs) were processed by GATK (v4.4.0) [17].

### 2.4. Acquisition and Analysis of Environmental Data

Sampling locations for different goose breeds were obtained from the location where the samples were collected; if the sampling location was not clear, the traditional geographic coordinates of the breed’s domestication would be taken according to a published book written by the China National Commission of Animal Genetic Resources [5]. Bioclimatic variables were taken from Worldclim (v2.1) [18,19] according to the longitude and latitude via ArcMap (v10.8). Worldclim is a high-resolution (30 arcseconds, about 1 km) dataset of regional climate on Earth’s land surface. It includes the average monthly and annual temperatures and precipitation patterns for 1970–2000. We extracted 19 climate factors from this dataset, including 11 variables related to temperature (unit: °C), the Annual Mean Temperature (bio1), Mean Diurnal Range (bio2), Isothermality (bio3), Temperature Seasonality (bio4), Max Temperature of Warmest Month (bio5), Min temperature of Coldest Month (bio6), Temperature Annual Range (bio7), Mean Temperature of Wettest Quarter (bio8), Mean Temperature of Driest Quarter (bio9), Mean Temperature of Warmest Quarter (bio10), Mean Temperature of Coldest Quarter (bio11), and 8 variables related to precipitation (unit: mm), Annual Precipitation (bio12), Precipitation of Wettest Month (bio13), Precipitation of Driest Month (bio14), Precipitation Seasonality (bio15), Precipitation of Wettest Quarter (bio16), Precipitation of Driest Quarter (bio17), Precipitation of Warmest Quarter (bio28), and Precipitation of Coldest Quarter (bio19) (Supplementary file S1: Table S1).

### 2.5. Genetic Diversity Analysis

FST is one parameter that can reflect the degree of population differentiation, which ranges from 0 to 1, where 0 indicates that the population is not differentiated, and 1 indicates that the population is completely differentiated. Based on all high-quality clean data, we calculated the pairwise F_ST_ [20] in 21 goose breeds using vcftools (v 0.1.16) [21]. The expected heterozygosity and observed heterozygosity estimates for all individuals within each breed were averaged using PLINK (version 1.9) [22]. Inbreeding coefficients can reflect the degree of animal inbreeding. Lengths of homozygous fragments were scanned using PLINK (version 1.9) [22] with --LROH parameter. Then, inbreeding coefficients of each sample and breed were calculated as the ratio of the length of runs of homozygosity and the length of the autosomal genome spanning the SNP positions (984.24 kb in this study) [23].

### 2.6. Population Structure and Phylogenetic Analysis

According to the parameter --geno 0.1 --maf 0.1 --indep-pairwise 20 10 0.5, SNPs were filtered using PLINK (v1.90) [22]. Sites on sex chromosomes were removed, yielding a total of 2,638,406 independent autosome SNPs that were used for subsequent analysis. The PCA analysis was also performed by PLINK (v1.90), and the unrooted Maximum likelihood tree was constructed using SNPhylo (v20180901) [24]. The tree visualization was achieved through the Interactive Tree of Life (ITOL 7.1.1, https://itol.embl.de). Population structure analysis was performed using ADMIXTURE (v1.3.0) software [25] with default parameters. The predefined genetic clusters (K) ranging from 2 to 10 to cover the maximum number of lineages, and the best-fit number of K was considered to hold the smallest cross-validation error (CV error) value.

### 2.7. Detection of SNPs Associated with Environmental Adaptations

C-program-based R package LFMM (v1.5) [26] was used to calculate correlations between climate variables and SNPs. LFMM aims to screen genomes for signatures of environmental adaptation. To correct the confounding effects, K = 6, which holds the lowest CV error based on the results of admixture analysis, was set as the number of latent factors. SNPs with corrected *p* < 0.01 were considered to be the candidate sites associated with the corresponding climate variable. Since LFMM is a univariate detection method, we further applied two multivariate landscape genome methods, the individual spatial analysis and redundancy analysis (RDA).

Samβada (v0.8.3) [27] was used to detect correlations between genotypes and environmental variables. It aims to detect local adaptation in relation to the environment and the measure of spatial autocorrelation in environmental and molecular datasets by calculating multiple univariate logistic regression analyses. Log-likelihood (G) test and Wald test were used to determine the significance of the association. Bonferroni correction is applied to correct for multiple comparisons.

RDA is also a multivariate method [28] to identify the genetic variation loci associated with environmental variables. Before RDA analysis, environmental variables were assessed by ranked accuracy importance and the Pearson correlation method. Gradient forest (GF) analysis was performed to identify 19 environmental variables that best explained the distribution of genetic variation using the R package gradientForest (v0.1-37) [29] based on all SNPs with default parameter values. Furthermore, the absolute value of Pearson correlation coefficients among 19 environmental variables was evaluated using R. The R package “vegan” [30] was used for RDA analysis with genetic variation set as the response variable, climate variables set as explanatory variables, and latitude, longitude, and population structure effects set as covariates.

### 2.8. Functional SNPs Detection Approach

To further assess selection pressures acting on temperature and precipitation adaptive variants, we first calculated the allele frequencies of all candidate SNPs using vcftools (v0.1.16) [21], and selected a set of variants that the allele frequencies exhibit regular changes with temperature or precipitation in different breeds. Then, the extended haplotype homozygosity (EHH) for variants in the selected set was calculated using the R package “rehh” [31] to further search for footprints of selection. The EHH value reflects the degree of homozygosity of the haplotype around a certain SNP locus in the population. When a SNP locus is under positive selection, the genetic diversity of the surrounding region linked to it will decrease, and the EHH value will increase. This is because the selected allele will spread rapidly in the population, and the haplotype carrying this allele will become more common, resulting in a relatively higher degree of homozygosity of the haplotypes in the surrounding region.

### 2.9. Gene Annotation and KEGG Analyses

In order to annotate candidate SNPs and obtain official names for protein-coding genes, we further improved the reference genome annotation file by direct homologous protein alignment using eggnog (evolutionary genealogy of genes: Non-supervised Orthologous Groups) [32] (Supplementary file S1: Table S2). The filtering parameter was set as “--Minimum hit e-value 0.001, --Minimum hit bit-score 60, --Percentage identity 40, --Minimum % of query coverage 20, --Minimum % of subject coverage 20”. KEGG pathway analysis of candidate genes was performed by David [33,34].

## 3. Results

### 3.1. Sequencing Mapping Quality and Variant Discovery

Across all samples, a total of 1779.5 million mapped reads were obtained, with an average genome depth of 9.95×, and an average coverage of 97.8% (Supplementary file S1: Table S3). Sequences were mapped to the reference genome of goose with an average mapping ratio of 98.07% (Supplementary file S1: Table S3). After variant calling, a total of 8.81 million variants were obtained, including 6.67 million SNPs and 1.14 million indels.

### 3.2. Population Genetic Diversity of Chinese Geese

The genetic differentiation (F_ST_ values) pairwise estimates between 21 goose populations are shown in Supplementary file S1: Table S4. Genetic differentiation had occurred among different varieties. The average F_ST_ values ranged from 0.04 to 0.91. The highest F_ST_ 0.91 is between ZJ goose and MB goose. The lowest F_ST_ 0.04 is between TH and SC goose, next is between ZI and HY goose. The expected heterozygosity, observed heterozygosity, and the genomic inbreeding coefficient (F_ROH_) of each breed were shown in Supplementary file S1: Table S5. The expected heterozygosity of SC (0.323) and TH (0.312) populations was the highest, followed by the HY (0.309) goose, MG goose (0.307), and ZI goose (0.304). A comparison of the F_ROH_ across the 21 populations showed that F_ROH_ of YL (0.262) and MB (0.262) goose were the highest, followed by ZI goose (0.255) and HY goose (0.227).

### 3.3. Population Genomic Divergence

**Figure 2:**
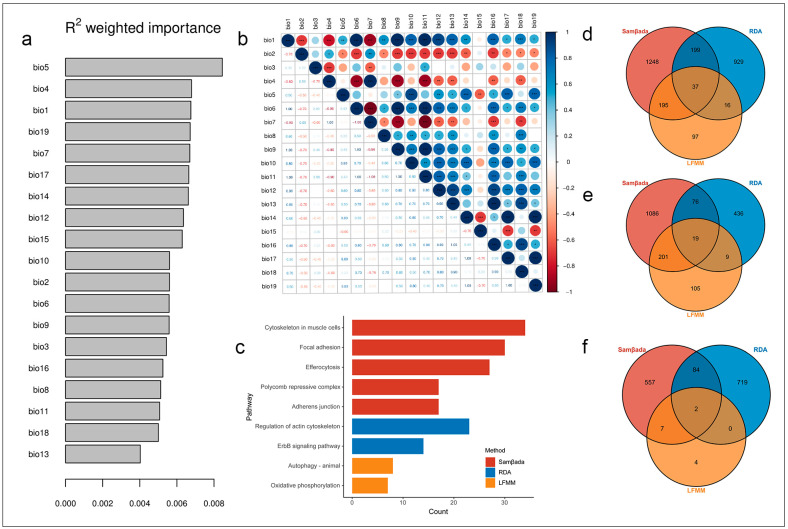
(**a**). Ranked importance of environmental variables based on GF modelling. (**b**). Pearson correlation between 19 climate factors. Notably, the * typically denotes significance at the 0.05 level (*p* ≤ 0.05), ** indicates significance at the 0.01 level (*p* ≤ 0.01), and *** represents significance at the 0.001 level (*p* ≤ 0.001). (**c**). Significant KGEE pathways enriched based on candidate genes of three GEA methods. (**d**). Venn plot of the gene number of three GEA methods. (**e**). Venn plot of the temperature-associated genes selected by three GEA methods. (**f**). Venn plot of the precipitation-associated genes selected by three GEA methods.

The phylogenetic relationships between individual samples were inferred based on the ML trees constructed (Figure 2b). In the genome-wide tree, all samples formed six clusters. Firstly, the MG and YANGJ clustered, and then, the XG, XP, DZ, YOUJ, and ST clustered. Next, the FC, GS, GE, CL, YK, ZD, MB, and GFW formed one cluster. The YL goose clustered independently, while two breeds (the HY and ZI) from northern China formed another clade. Finally, TH and SC goose clustered with several XP and ZI geese.

The PCA analysis was performed with the first two PCs accounting for 58.16% of the total genetic variation using 2,638,406 SNPs (45.38% and 12.78% for PC1 and PC2, respectively; Figure 2c). In the first principal component, the YL goose first separated from other breeds, and then breeds from southern China roughly formed another three clusters: the YANGJ, MG, XG, XP, DZ, YOUJ, and ST; the FC, GS, GE, CL, YK, ZD, MB, and GFW; and the TH and SC. Finally, two breeds from northern China, the ZI goose and HY, were grouped together. The clustering observed supported the ML tree above.

Further, the population structure was inferred using ADMIXTURE to deduce the population admixture proportions by assigning ancestral populations K from 2 to 10. Part of the results are shown in Figure 2d. At K = 2, YL goose was isolated from all other breeds. The ZI goose and HY goose were separated subsequently at K = 3. At K = 6 was identified the most likely number of evolutionary clusters among the 21 breeds with the lowest CV error (0.6268, Supplementary file S2: Figure S1). The six clusters included are, in turn, the ZI and HY, the SC and TH, the ST, the YANGJ and MG, the YL, and the remaining breeds formed two clusters with complex ancestral components (YOUJ, CL, DZ, FC, GE, GFW, GS, MB, XG, YK, ZD, ZJ, XP).

### 3.4. Identification of Genomic Variants Associated with Local Climate Adaptability

Based on the univariate approach LFMM method, we identified 3994 SNPs (Supplementary file S1: Table S6) that were significantly associated with one or more climate variables. Within ±10 kb regions of these selected SNPs, 345 genes were annotated (Supplementary file S1: Table S7). These variants were widely distributed across the genome, but notably, 854 SNPs (21.38%) were located on chromosome 3. Three significant KEGG pathways (*p* < 0.05) were enriched, including oxidative phosphorylation (acyg00190), autophagy-animal (acyg04140), and polycomb repressive complex (acyg03083) (Figure 2c).

Samβada is an integrated software for landscape genomic analysis of large datasets. The key features are the study of local adaptation in relation to the environment and the measure of spatial autocorrelation in environmental and molecular datasets. A total of 150,389,161 univariate models (7,915,219 genotypes × 19 environmental variables) were used in the analyses. We selected the top 16,000 models (Supplementary file S1: Table S8) based on Wald scores for further analysis, accounting for approximately 0.01% of all univariate models. The most common correlated environmental variables included bio10 (4344 models, 27.15%), bio5 (3576 models, 22.35%), bio19 (1084 models, 6.78%), and bio14 (1005 models, 6.28%). A total of 7573 SNPs were found to be under selection, and within the ±10 kb regions of these selected SNPs, we annotated 1679 known functional genes (Supplementary file S1: Table S9). These genes were significantly enriched in five KEGG pathways involving the cytoskeleton in the muscle cells pathway (acyg04820), focal adhesion pathway (acyg04510), efferocytosis pathway (acyg04148), polycomb repressive complex pathway (acyg03083), and adherens junction pathway (acyg04520) (Figure 2c).

According to the results of GF analysis, the top three temperature variables identified by GF are bio5, bio4, and bio1, and the top three temperature variables identified by GF are bio19, bio17, and bio14 (Figure 2a). In terms of the Pearson correlation analysis, to address the multicollinearity issue, the set of climate variables exhibiting high correlations (|r| > 0.7) was reduced by retaining only one representative variable (Figure 2b). Taking the results of both GF and Pearson correlation analysis into account, six climate variables, including three temperature variables (bio2, bio4, and bio5) and three precipitation variables (bio12, bio15, and bio19), were used for subsequent RDA analysis. The RDA model constructed is significant (*p* < 0.01), and 2.95% of total genetic variations can be explained. For the six significant constrained axes, the total genetic variance of RDA was from 9% to 34% (Supplementary file S2: Figure S2). A total of 9967 SNPs were found to display extreme loadings (standard deviation > 3.5) along one or multiple RDA axes (Supplementary file S1: Table S10), and 1461 genes were obtained (Supplementary file S1: Table S11). Two pathways were enriched, including the ErbB signalling pathway (acyg04012) and the regulation of actin cytoskeleton pathway (acyg04810).

Finally, 447 genes were selected by at least two methods (Supplementary file S1: Table S12). Taking these genes as input, three pathways were significantly enriched, including the polycomb repressive complex pathway (acyg03083), the alanine, aspartate, and glutamate metabolism pathway (acyg00250), and the GnRH signalling pathway (acyg04912) (Supplementary file S1: Table S13). Further filtering suggested 37 genes were identified by all 3 methods (Figure 2d, Supplementary file S1: Table S14), 19 genes (*RALY*, *EPYC*, *SLCO4A1*, *SDCCAG8*, *EEF1A2*, *RAB11FIP3*, *GPC3*, *TMEM104*, *STX17*, *GTF2IRD1*, *FBRSL1*, *RCAN2*, *GFRA1*, *C14orf80*, *SLC22A15*, *GPR149*, *DHX36*, *SCN8A*, *EXOC4*) were temperature associated (Figure 2e, Supplementary file S1: Table S15), and 2 genes (*EXOC4*, *USP13*) were precipitation associated (Figure 2f, Supplementary file S1: Table S16). Notably, the *EXOC4* gene was the only one identified that was associated with both temperature and precipitation.

### 3.5. SNPs Associated with Environmental Variables

**Figure 3:**
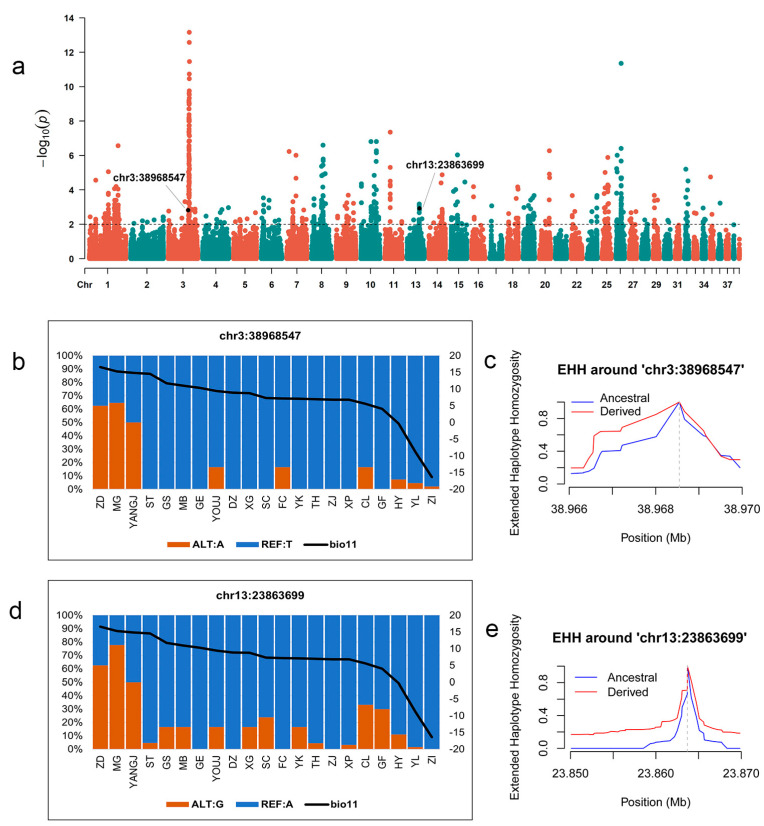
(**a**) Manhattan plot for variants associated with the Mean Temperature of Coldest Quarter (bio11). (**b**) Allele frequencies of chr3:38968547. The T allele is represented in blue, and the A allele is represented in orange. The black lines correspond to Mean Temperature of Coldest Quarter. (**c**) Extend haplotype homozygosity around chr3:38968547. (**d**) Allele frequencies of chr13:23863699. The A allele is represented in blue, and the G allele is represented in orange. The black lines correspond to Mean Temperature of Coldest Quarter. (**e**) Extend haplotype homozygosity around chr13:23863699.

The frequency of SNPs located in the CDS region of 447 candidate genes was calculated first; however, these SNPs did not show a similar change as the climate variables of the 21 breeds. Further analysis concentrated on these SNPs, which were selected by three methods, and finally, two SNPs located in the non-coding region were identified (Figure 3a). One is chr3:38968547, and the other is chr13:23863699. SNP chr3:38968547 is located between the OR52B2 and PRSS23 genes. SNP chr3:38968547 is located between the *HAL* and *AMDHD1* genes. The mutated allele A of chr3:38968547 and allele G of chr13:23863699 were mainly distributed in the region with higher temperature (bio11), whereas their alleles T and A were almost fixed in areas experiencing lower temperature (Figure 3b,d). EHH results show that both SNPs experienced strong positive selection (Figure 3c,e).

## 4. Discussion

In this research, we utilized 239 genome sequencing data from a vast number of Chinese local goose breeds, uncovering 70% of Chinese local goose breeds. About 2.64 million clean SNPs were obtained to uncover the population structure of these Chinese local geese. Results of the genetic structure suggested that Chinese goose breeds can be roughly divided into six clusters based on geographical location. Four clear clusters are as follows: the YL, the ZI and HY, the SC and TH, and the MG and YANGJ. This can be explained by the results in the genetic diversity Section that breeds formed together have lower F_ST_. Another two clusters consisted of breeds all from Southern China. These breeds were geographically proximate yet exhibited intricate ancestry components, suggesting a complex genetic history and potentially extensive gene flow among them. One includes the XG, XP, DZ, YOUJ, and ST goose, while another one includes the FC, GS, GE, CL, YK, ZD, MB, and GFW goose. In the phylogenetic and PCA analyses, and at K = 2, the YL goose was separately clustered with all other breeds, which is possibly because the Yili goose has a different ancestor from other goose breeds [3]. High inbreeding coefficients (exceeding 0.2) observed in 12 goose breeds, namely, GE, HY, MB, MG, SC, ST, TH, YANGJ, YK, YL, ZI, and ZJ, further indicate the widespread occurrence of admixture events and inbreeding within Chinese local goose populations. This concerning situation underscores the urgency of implementing additional conservation measures to safeguard these valuable genetic resources. For example, maintaining an effective population size of each breed, implementing controlled mating strategies to prevent consanguineous pairings, introducing non-related genetic material from other populations or lines, and establishing a conservation and genetic monitoring program to track genetic diversity over time.

Among 447 candidate genes, the *FAF1* and *RSF1* genes were candidates for cold adaptation in Chinese geese [12]. Eight members of the SLC family (*SLC6A14*, *SLC4A11*, *SLC38A7*, *SLC38A1*, *SLC36A4*, *SLC34A2*, *SLC22A15*, *SLC16A1*), which mainly perform a function of transporting various substrates across biofilms, including the absorption of small molecules into cells to maintain a constant internal environment, were selected [35]. Some members of this family have been identified as strongly associated with tropical environments (*SLC33A1*, *SLC13A1*, *SLC5A11*) [8,36]. Moreover, some other members, including *SLC7A7*, *SLC2A3*, *SLC16A1,* and *SLC16A7,* were proposed as having a high potential as heat stress biomarkers for different organisms [37,38]. These results reasonably supported that candidate genes belonging to the SLC family are important for temperature adaptation of Chinese geese. In addition, three members of the NADH dehydrogenase (ubiquinone) iron-sulfur protein family (*NDUFS5*, *NDUFB5*, *NDUFB2*), which plays an important role in energy metabolism [39], were selected. One of this family, the *NDUFS4* gene, was reported as being helpful for chickens to achieve rapid adaptation to frigid environments [8]. Furthermore, these three genes were enriched in the oxidative phosphorylation pathway in this study, which serves as a critical biochemical process that supplies the energy required for cellular function and is indispensable for sustaining cellular life activities [40]. It is worth noting that the *EXOC4* gene was identified with both temperature and precipitation. The *EXOC4* gene mainly plays a function on various phenomena, such as cell migration, invadopodia formation, cytokinesis, glucose uptake, and neural development [41,42], making it possible to participate in environmental adaptation for different goose breeds by regulating metabolism.

Scanning all candidate SNPs, we did not find functional ones in the CDS region that show similar variability patterns with corresponding temperature or precipitation changes, whereas two SNPs (chr3:38968547, chr13:23863699) associated with bio11 (Mean Temperature of Coldest Quarter) located in non-coding region that experienced stronger positive selection showed a similar variability pattern with the temperature or precipitation changes in different goose breeds. Similar results are also described in other studies, whereby SNPs in introns or regulatory regions were involved in the adaptation of organisms to the environment [6,43,44]. This reveals that the sequence variation in the non-coding region played an important role in the environmental adaptation of Chinese geese. Four genes, the *OR52B2*, *PRSS23*, *HAL*, and *AMDHD1*, were located around two specific variants. The *OR52B2* gene belongs to the olfactory receptor (OR) family 52, playing an important role in initiating neuronal responses and triggering olfactory perception [45], indicating that temperature may have a significant impact on the volatilization and spread of odor substances, making odors more perceptible. Goose breeds living in high temperatures may have a more sensitive sense of smell. Another three genes, the *PRSS23* [46], *HAL* [47], and *AMDHD1* [48] genes may be involved in environmental adaptation by participating in metabolism.

In summary, this study revealed the genetic diversity of Chinese local goose and offered valuable references for breeding. A range of environment association genes is identified, which may may lead to certain genetic approaches for goose breeding in the context of climate change. However, since environmental adaptation is a complex polygenic controlled process, how these genes specifically regulate populations to adapt to different environments and how these variants or genes are applied remains to be studied.

## 5. Conclusions

In conclusion, our study investigated the genetic diversity of Chinese local geese and revealed that more effective measures need to be taken to better protect them. Furthermore, a range of functional genes associated with the environmental adaptability of Chinese geese was identified through genetic–environment association methods, and two significant variants showing stronger positive selection in high-temperature environments were discovered. This research is helpful for deepening our understanding of the genetic basis of goose adaptation to the environment and also provides a valuable resource for future selective breeding programs in the goose industry.

## Data Availability

Whole-genome resequencing data of 89 Chinese domestic geese sequenced in this study are available from the corresponding author upon reasonable request.

## Supplementary Materials

The following supporting information can be downloaded at: https://www.mdpi.com/article/10.3390/ani15101395/s1, Figure S1: CV error values for different number of K from 2 to 10; Figure S2: Eigenvalues for six constrained axes; Table S1: Summary of sample and environmental factor values of 21 populations; Table S2: Gene annotation results based on reference gff file; Table S3: Quality of sequencing data; Table S4: Genetic differentiation between 21 goose breeds; Table S5: Heterozygosity and inbreeding coefficient of 21 goose breeds; Table S6: Candidate SNPs identified by LFMM; Table S7: Candidate genes identified by LFMM; Table S8: Candidate models identified by Samβada; Table S9: Candidate genes identified by Samβada; Table S10: Candidate SNPs identified by RDA; Table S11: Candidate genes identified by RDA; Table S12: Candidate genes at least identified by two methods; Table S13: KEGG pathways enriched by genes at least identified by two methods; Table S14: Candidate genes identified by all three methods; Table S15: Temperature associated genes identified by all three methods; Table S16: Precipitation associated genes identified by all three methods.

## Abbreviations

*FAF1*	Fas-Associated Factor 1
*RSF1*	Remodeling and Spacing Factor 1
*SLC6A14*	Solute Carrier Family 6 Member 14
*SLC4A11*	Solute Carrier Family 4 Member 11
*SLC38A7*	Solute Carrier Family 38 Member 7
*SLC38A1*	Solute Carrier Family 38 Member 1
*SLC36A4*	Solute Carrier Family 36 Member 4
*SLC34A2*	Solute Carrier Family 34 Member 2
*SLC22A15*	Solute Carrier Family 22 Member 15
*SLC16A1*	Solute Carrier Family 16 Member 1
*NDUFS5*	Ubiquinone Oxidoreductase Subunit S5
*NDUFB5*	Ubiquinone Oxidoreductase Subunit B5
*NDUFB2*	Ubiquinone Oxidoreductase Subunit B2
*EXOC4*	Exocyst Complex Component 4
GEA	Genetic–environment association
CL	Changle goose
DZ	Daozhou goose
FC	Fengcheng White goose
GE	Gang goose
GFW	Guangfeng White goose
GS	Gushi goose
MB	Minbei White goose
MG	Manggang goose
ST	Shitou goose
TH	Taihu goose
XG	Xingguo Grey goose
YangJ	Yangjiang goose
YK	Yongkang goose
YouJ	Youjiang goose
ZD	Zhedong White goose
ZJ	Zhijin goose
YL	Yili goose
HY	Huoyan goose
XP	Xupu goose
ZI	Zi goose
SC	Sichuan White goose
SNPs	Single-nucleotide polymorphisms
EHH	Extend haplotype homozygosity
CDS	Coding sequencing
