# Supplementary material for: Whole-Genome Sequencing Identifies Functional Genes for Environmental Adaptability in Chinese Geese
Source: Animals (Basel). 2025 May 12;15(10):1395. doi: 10.3390/ani15101395 (PMC12108252; doi:10.3390/ani15101395)
Supplement: Supplementary file 1 [file animals-15-01395-s001.zip › supplementary file S2 Figures.pdf]

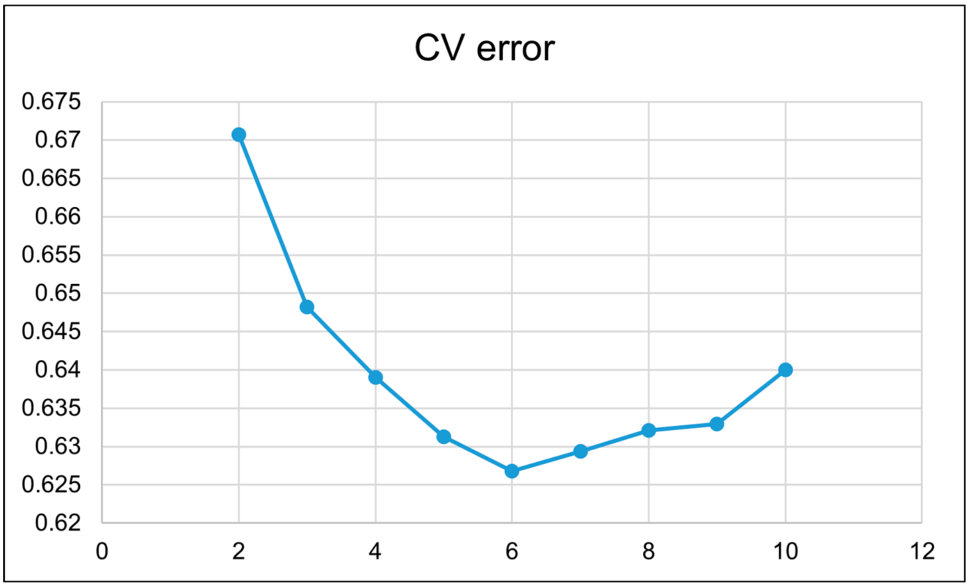

Figure S1 CV error values for different number of K from 2 to 10.

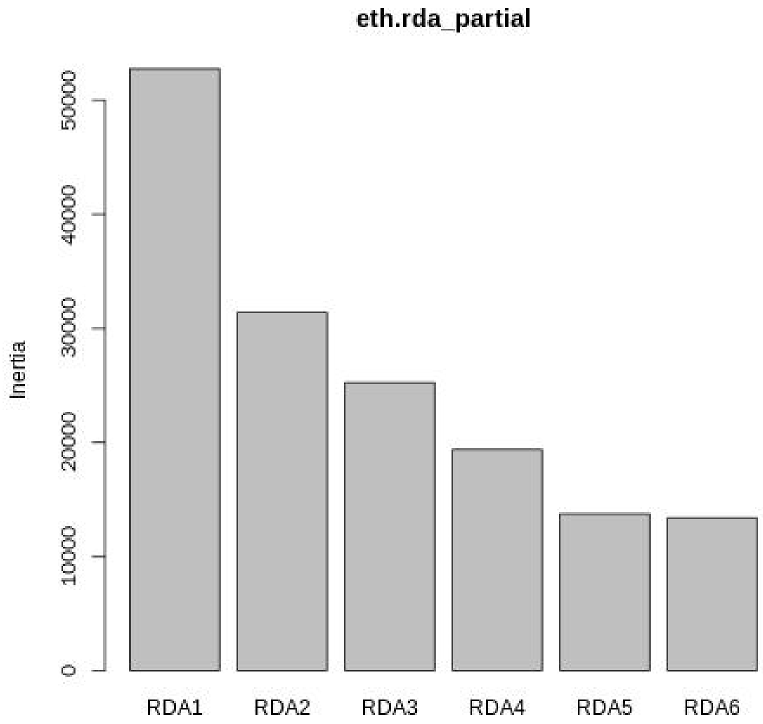

Figure S2 Eigenvalues for constrained axes.
